# Authors’ Reply: The Importance of Comparing New Technologies (AI) to Existing Tools for Patient Education on Common Dermatologic Conditions: A Commentary

**DOI:** 10.2196/72540

**Published:** 2025-04-01

**Authors:** Courtney Chau, Hao Feng, Gabriela Cobos, Joyce Park

**Affiliations:** 1 Icahn School of Medicine at Mount Sinai, New York, NY, United States; 2 Department of Dermatology, University of Connecticut Health Center, Farmington, NY, United States; 3 Department of Dermatology, Tufts Medical Center, 260 Tremont St, Fl 13, Bostom, MA, 02116, United States, 1 617-636-0156; 4 Skin Refinery PLLC, Spokane, WA, United States

**Keywords:** artificial intelligence, ChatGPT, atopic dermatitis, acne vulgaris, actinic keratosis, rosacea, AI, diagnosis, treatment, prognosis, dermatological diagnoses, chatbots, patients, dermatologist

Juels Parker commented on our study comparing the sufficiency of ChatGPT, Google Bard, and Bing artificial intelligence (AI) in generating patient-facing responses to questions about five dermatological diagnoses [1,2]. He highlights an important need to compare AI to existing patient education tools, such as handouts, peer-reviewed articles, and patient-centered websites.

We agree that AI is not a benign entity, and many resources exist for patients to learn about their conditions, aside from AI [3,4]. We also agree that AI cannot be deemed superior to existing materials without a comparative assessment. Yet, inherent differences between AI and existing materials inhibit such comparison in the context of our original study.

Our pilot study compares AI chatbot responses to potential patient questions, with the primary goal of comparing the utility of three chatbots by assessing their strengths and weaknesses. As suggested by Parker, recommending the usage of AI in place of existing patient education materials would require a larger, more robust investigation that compares AI to existing resources. In our study, however, AI plays an inherently different role than traditional patient resources, such as paper handouts, disallowing comparative assessment. Generative AI offers users the flexibility to ask questions and receive direct answers, whereas traditional forms of patient education require patients to search for answers to their questions. By evaluating generative AI, our study simulates how patients might ask questions in the real world. As such, a comparison to existing patient resources was out of the scope of our study and would not have answered our research question—to evaluate the utility of chatbots to generate patient-facing responses. Additionally, patient education materials vary between practices, hindering the ability to conduct a comparative analysis with applicability real practice. While our conclusions suggest that AI may be used by patients to obtain information about their condition, we emphasize that this recommendation is to a limited extent and chatbots should not function as a first-line entity. Only approximately half of the responses in our study were considered sufficient for clinical practice, highlighting three domains in which chatbots require improvement—readability, removing inaccuracies, and improving specificity.

In conclusion, Parker highlights an important consideration regarding AI in dermatology–whether information gleaned from AI is superior to existing patient resources. However, in the context of our study, a comparative analysis between AI and existing resources would not have contributed to our goal of comparing chatbots. In the broader context of AI in dermatology, a study with a primary intention of comparing AI and existing materials for their clinical utility would provide novel insights into the future of AI in practice. Our pilot study is not sufficient to and does not confidently recommend patient usage of AI. Rather, our study serves as a basis for further examination of AI’s role in dermatology by illustrating the strengths and weaknesses of different chatbots. We appreciate the critical thought that Parker discussed about the implications of our work and the role of AI in dermatology.

## Abbreviations

AI: artificial intelligence
